# Structure-Function Relation of Phospholamban: Modulation of Channel Activity as a Potential Regulator of SERCA Activity

**DOI:** 10.1371/journal.pone.0052744

**Published:** 2013-01-04

**Authors:** Serena Smeazzetto, Andrea Saponaro, Howard S. Young, Maria Rosa Moncelli, Gerhard Thiel

**Affiliations:** 1 Dipartimento di Chimica, Università di Firenze, Firenze, Italy; 2 Department of Biology and CNR IBF-Mi, Università degli Studi di Milano, Milano, Italy; 3 Department of Biochemistry, University of Alberta, Edmonton, Canada; 4 Plant Membrane Biophysics, TU-Darmstadt, Darmstadt, Germany; Virginia Commonwealth University, United States of America

## Abstract

Phospholamban (PLN) is a small integral membrane protein, which binds and inhibits in a yet unknown fashion the Ca^2+^-ATPase (SERCA) in the sarcoplasmic reticulum. When reconstituted in planar lipid bilayers PLN exhibits ion channel activity with a low unitary conductance. From the effect of non-electrolyte polymers on this unitary conductance we estimate a narrow pore with a diameter of ca. 2.2 Å for this channel. This value is similar to that reported for the central pore in the structure of the PLN pentamer. Hence the PLN pentamer, which is in equilibrium with the monomer, is the most likely channel forming structure. Reconstituted PLN mutants, which either stabilize (K27A and R9C) or destabilize (I47A) the PLN pentamer and also phosphorylated PLN still generate the same unitary conductance of the wt/non-phosphorylated PLN. However the open probability of the phosphorylated PLN and of the R9C mutant is significantly lower than that of the respective wt/non-phosphorylated control. In the context of data on PLN/SERCA interaction and on Ca^2+^ accumulation in the sarcoplasmic reticulum the present results are consistent with the view that PLN channel activity could participate in the balancing of charge during Ca^2+^ uptake. A reduced total conductance of the K^+^ transporting PLN by phosphorylation or by the R9C mutation may stimulate Ca^2+^ uptake in the same way as an inhibition of K^+^ channels in the SR membrane. The R9C-PLN mutation, a putative cause of dilated cardiomyopathy, might hence affect SERCA activity also via its inherent low open probability.

## Introduction

The contraction and relaxation of the heart is controlled by a periodic increase and decrease of the Ca^2+^ concentration in the cytosol [1]. During relaxation of the cardiac myocytes the sarco-endoplasmic CaATPase (SERCA) maintains a low cytosolic calcium concentration by pumping Ca^2+^ into the lumen of the sarcoplasmic reticulum (SR) in exchange with lumenal H^+^ [2]. SERCA is regulated by phospholamban (PLN), an integral membrane protein of only 52 amino acids (AA), which is composed of three domains: domain IA, a helical cytoplasmic domain (AAs 1–16), domain IB, a semi-flexible loop (AAs 17–21) and domain II, a helical hydrophobic transmembrane domain (AAs 22–52) [3], [4]. The activity of SERCA is inhibited by un-phosphorylated PLN whereas phosphorylated PLN releases SERCA inhibition and allows pumping of Ca^2+^. PLN can be phosphorylated at different residues in domain I: Ser-10 by protein kinase C, Ser-16 by cAMP-dependent protein kinase and Thr-17 by Ca^2+^-calmodulin dependent kinase [1].

PLN occurs as a monomer (6 KDa) and as a pentamer (30 KDa) and both oligomeric forms are in an equilibrium [5]. While it is clear that monomeric PLN is sufficient for SERCA inhibition, the functional relevance of the oligomeric states is not yet fully understood. [6], [7], [8]. Also structural details of the PLN pentamer and in particular the question whether it forms a central ion conducting pore, are not yet clarified. Currently a so called “bellflower” [9] and a “pinwheel” structure [4] of the pentamer are discussed as structural models in the literature [4], [9], [10], [11]; some structural data support [9], [10], [11] and others reject [4] the idea that a central pore in the pentamer can serve as pore for ionic currents.

In previous work it has been observed that PLN generates single channel fluctuations between a closed and two defined open states with distinctly different conductances [12], [13]. The channel has a moderate cation selectivity between similar cations K^+^ and Na^+^; under the conditions tested it exhibits no appreciable permeability to the larger cations Ca^2+^ or choline (Cho^+^) [12]. The results of these experiments foster the hypothesis that the activity of the channel can in principle be regulated in the physiological context on the basis of a modulated open probability. In this way PLN can generate a short circuit that locally modifies the electrochemical gradient across the SR membrane.

To gain more information on structure/function correlates of PLN in relation to channel formation we tackle here the question whether channel activity is indeed generated by the pentamer or any other oligomeric form of the protein. Furthermore we analyse selected mutants which are thought to stabilize the pentamer form (R9C [14], [15] and K27A [16]) or which are considered to be predominantly monomeric (I47A [17], [18]) in SDS electrophoresis gel. This analysis is in particular interesting in relation to the natural R9C-PLN dominant mutant in humans, which seems to be involved in several cases of familial dilated cardiomyopathy [1]. As yet the molecular mechanism which is underlying this disease is still not understood [15], [19]. In the context of the finding that PLN generates channel activity it is plausible that the disease phenotype is related by an aberrant channel activity of the PLN mutant and/or by an altered activity in the R9C-PLN/SERCA2a complex.

For the analysis of structure/function correlates we reconstituted wild-type (wt) PLN and its mutants in traditional black lipid membranes (BLMs) and monitored single channel activity. The data are in good agreement with the hypothesis that channel activity is indeed generated by the PLN in the pentamer form. Stabilization or destabilization of the pentamer does not *a priori* affect the unitary channel conductance [8], [18], [20]; a modulation of pentamer stability by phosphorylation and particular mutants of PLN however alters the open probability, another important functional property of the channel.

## Results and Discussion

**Figure 1:**
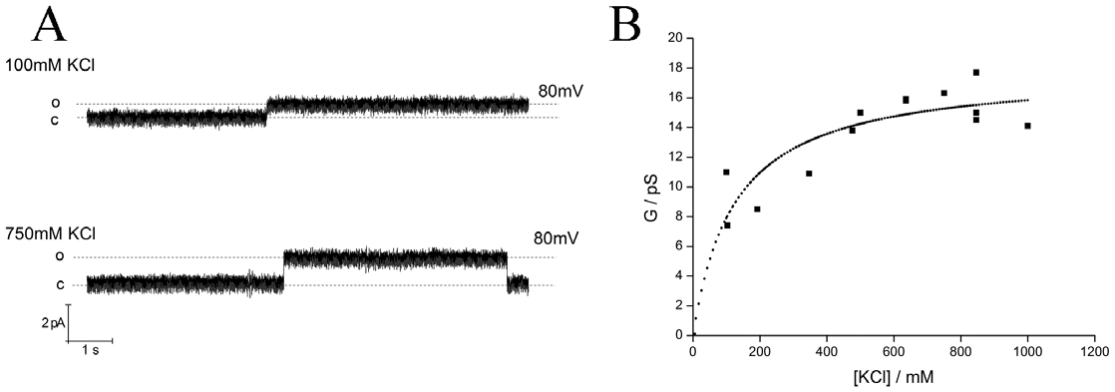
Titration curve: unitary channel conductance vs KCl concentration. (A) Example of current fluctuations measured at +80 mV in symmetrical solution with 100 mM or 750 mM KCl in 10 mM MOPS (pH = 7) buffer. (B) Titration of unitary channel conductance generated by PLN as a function of the KCl concentration. Experiments were done in symmetrical solution with KCl at increasing concentration in 10 mM MOPS (pH = 7) buffer. Fit of data with equation (1) yields concentration for half maximal conductance at 125 mM and a maximal conductance of 18 pS.

In order to characterize structure-function correlations of PLN the purified recombinant protein was reconstituted in traditional BLM. The canonical channel activity with currents fluctuating between well defined closed and open levels were recorded as reported in a previous paper [12]. For the present analysis we only use the larger of the two conductances. Figure 1A shows exemplary current fluctuations of the same channel at +80 mV in buffers with two different KCl concentrations. The open channel current increases as a function of the KCl concentration. The unitary channel conductance exhibits saturation kinetics (Figure 1B), which can be fitted with a Michaelis-Menten type equation; the fit provides a value of 125 mM for half maximal conductance and a maximal conductance G_max_ of 18 pS. The maximal value is similar, albeit a bit smaller, to the PLN generated conductance reported previously [12] probably due to the different lipid used to form the bilayer.

In a first approximation we estimated the pore size of the PLN generated channel using an Ohmic model for conductance [24] and considering a correction factor introduced by Smart et al. [25]. The latter authors have demonstrated that the conductance of the electrolyte solution within the pore is 5 times smaller than in solution. Using equation 1 with 12 pS as unitary conductance (Figure 1B) and a reasonable value of 28 Å for the length of the constriction zone [9] we can estimate a pore diameter of 3.4 Å. This value is within the range of diameters (2–8 Å) reported for the pore in the centre of the PLN pentamer [9], [28], [29], [30]. Hence the experimental data are in agreement with the hypothesis that the PLN pentamer generates channel fluctuations.

**Figure 2:**
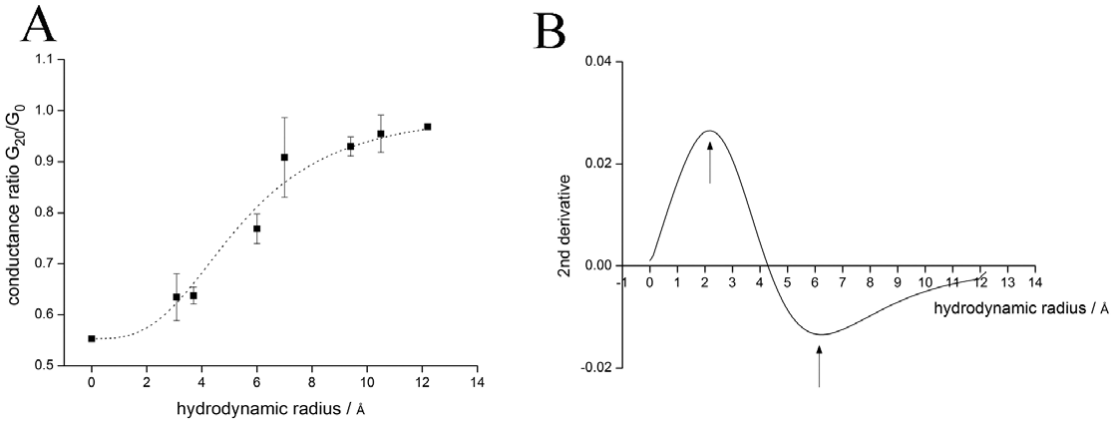
Estimation of pore size by means Polyethylene glycol method. (A) Normalized unitary channel conductance of PLN channel as a function of the hydrodynamic radius of polymers in buffer solution. Error bars indicate the standard error of the mean considering 4 independent experiments. (B) Second derivative of the fit in A. Arrows indicate the minimum and the maximum pore radius respectively.

To gain further information on the pore size we used the polymer exclusion method [26]. This method takes advantage of the fact that soluble non-electrolytes lower the diffusion coefficient of ions in solution and hence reduce the bulk conductance. When the size of the polymers exceeds a critical dimension it is excluded from the pore of a channel with the effect that its negative impact on the diffusion coefficient of the transported ion is decreased. A quantitative relationship between channel conductance and polymer size hence provides information on the size of a channel pore [25]. The experimental data in Figure 2 show the unitary channel conductance of the PLN channel as a function of the hydrodynamic radius of small non-electrolytes in the test solution (Figure 2A). The decrease in unitary conductance as a function of the hydrodynamic radius of the polymers is well fitted by a logistic equation. According to Smart et al. [25] the maximum and minimum of a second derivative (Figure 2B) of the fitted curve in Figure 2A provide the approximate radius for the most narrow part and the wider pore mouth, respectively. The respective values of 2.2 Å and 6.2 Å for the most narrow part and the wider part of the pore, which we estimated from our experimental data, are similar to the diameters found in the NMR structure and by molecular modelling of pentameric PLN; published data report pore size of ca. 3.6 Å [9], between 2 Å and 8 Å [28], ca. 2.5 Å [29] and ca. 3 Å [30]. The good agreement between experimental and structural data again supports the hypothesis that the conducting channel is indeed generated by the pentamer.

**Figure 3:**
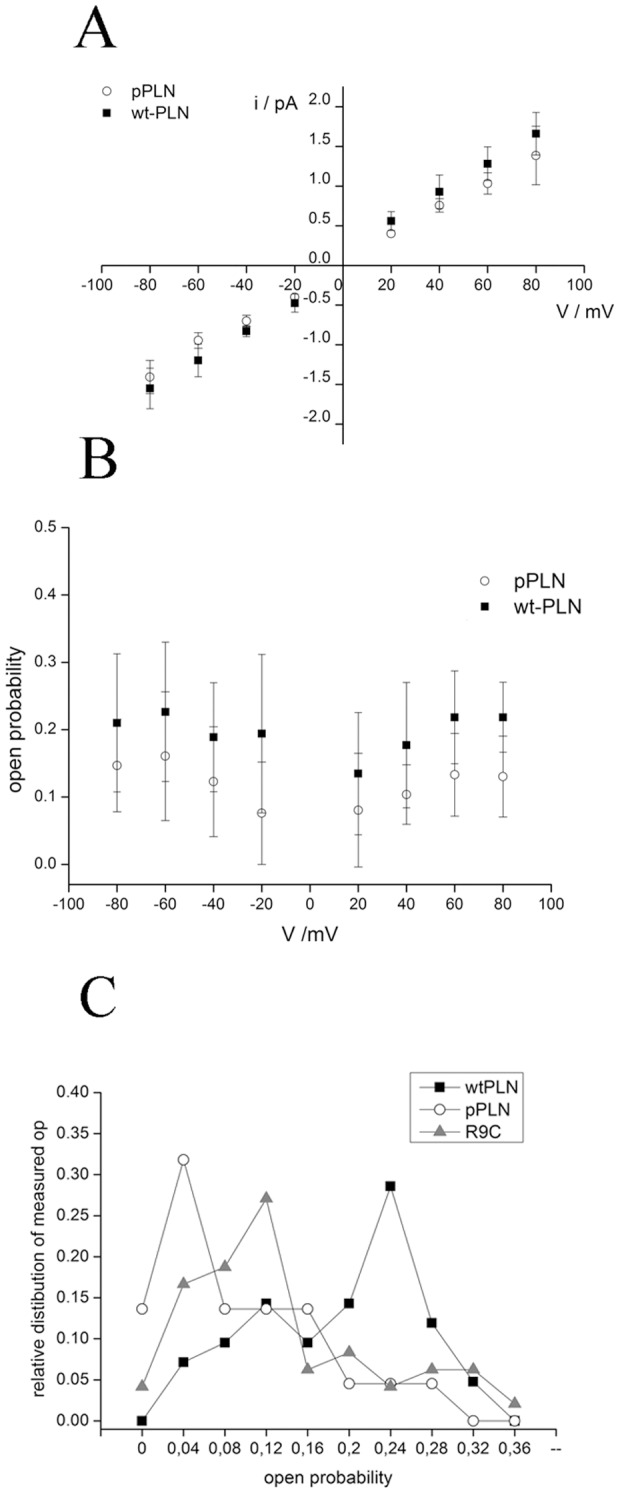
Effects of Phosphorylation on Phospholamban forming channel activity. (A) Current/voltage relation of unitary channel currents generated by phosphorylated (open circles) and non-phosphorylated wt-PLN (full squares) in symmetrical solutions with 500 mM KCl in 10 mM MOPS (pH = 7) buffer. Data are mean ± standard deviation (SD) of 10 independent experiments for the wt-PLN and 4 independent experiments for the pPLN. (B) The mean open probability Po (±SD) of phosphorylated (open circles) and non-phosphorylated PLN (full squares) obtained in symmetrical solutions with 500 mM KCl in 10 mM MOPS (pH = 7) buffer. The mean was calculated on the basis of 8 and 5 independent experiments with the wtPLN and pPLN, respectively. (C) Relative distribution of measured open probabilities for wtPLN (black) phosphorylated wtPLN (gray) and for R9C mutant (white) of wtPLN. Data are normalized for the total number of recordings.

In further experiments we examined whether phosphorylation of PLN, which is functionally significant for the regulation of SERCA [31], [32], has an impact on channel activity. For this purpose we compared single channel recordings of phosphorylated and non-phosphorylated PLN. We found that both forms of the protein exhibit the same prevailing unitary conductance of 18 pS; the I/V relations are quasi identical (Figure 3A). While the channel conductance is unaffected by phosphorylation, the open probability of the phosphorylated protein is appreciably lower than that of the non-phosphorylated form (Figure 3B). The open probability of the non-phosphorylated protein shows a broad distribution with a maximum at ca. Po = 0.25 (Figure 3C) and a mean value of 0.21±0.08 (n = 50 recordings). As a result of phosphorylation the open probability distribution shifts to lower values with a maximum at about Po = 0.04 and mean at 0.1±0.07 (n = 24 recordings). A Kolmogorov-Smirnov test indicates that the mean open probability of the phosphorylated form is significantly lower than that of the non-phosphorylated one with a value of P = 0.002.

**Figure 4:**
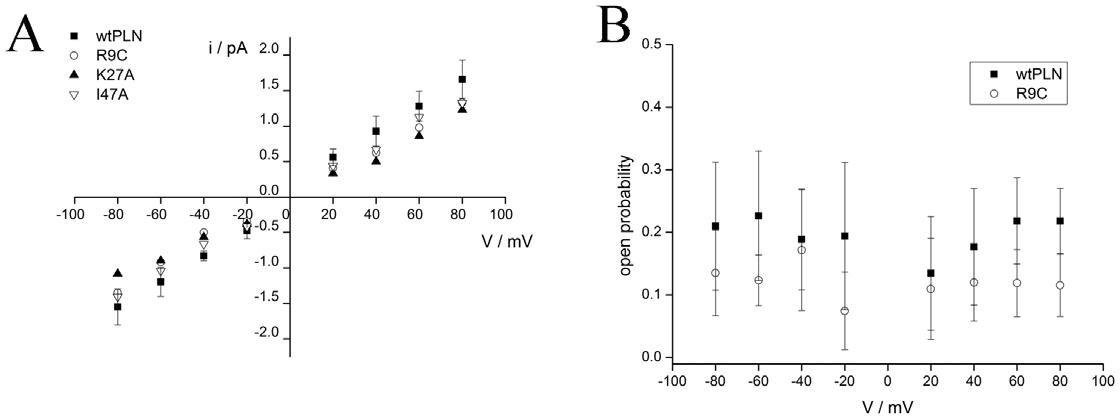
Effects of 3 selected PLN mutants (R9C, K27A, I47A) on channel activity. (A) Current/voltage relation of unitary channel currents generated by wt-PLN (full squares), K27A (full triangles), I47A (open triangles) and R9C (open circles); all data recorded in symmetrical solutions with 500 mM KCl in 10 mM MOPS (pH = 7) buffer. Data are mean ± standard deviation (SD) of 10 independent experiments for the wt-PLN and a minimum of 9 recordings for each of the 3 mutants. (B) The mean open probability (±SD) of wt-PLN (full squares) and R9C mutant (open circles) obtained in symmetrical solutions with 500 mM KCl in 10 mM MOPS (pH = 7) buffer. The mean was calculated on the basis of 8 independent experiments for both the wtPLN and the R9C mutant.

Previous experiments have shown that some PLN mutants can exhibit a different stability of the pentamer in SDS gels [15], [16], [18]. Measurements with the respective PLN mutants with different pentamer stability show that all of them exhibit the same prevailing unitary conductance of 18 pS known from the wt channel (Figure 4A). Hence stabilization or destabilization of the pentamer does not affect the unitary channel conductance. This results is in agreement with data obtained by cryo-electron microscopy studies [8] and by FRET measurements [20]. Again we noticed that the R9C mutant exhibited consistently a lower open probability (Po) than the wt channel in symmetrical KCl conditions (Figure 4B); the difference in open probability is according to a Kolmogorov-Smirnov test significant (P = 0.01).

**Figure 5:**
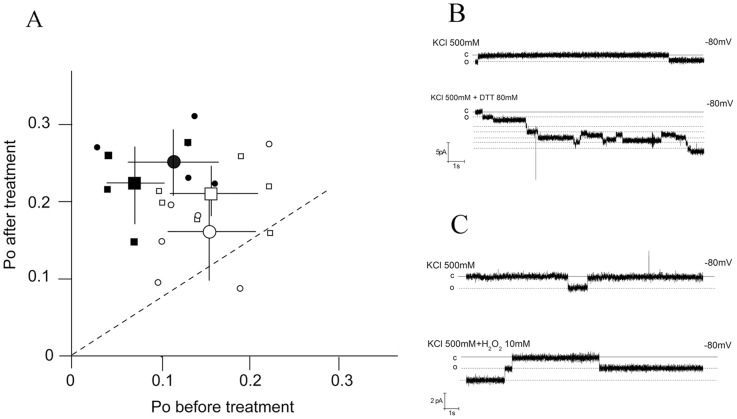
Increase of open probability in R9C mutant in reducing/oxidizing conditions. (A) Scatter plot of open probability for wtPLN (open symbols) and R9C mutant (filled symbols) before (x-axis) and after adding (y-axis) 80 mM DTT (circles) or 10 mM H_2_O_2_ (squares) to *trans* and *cis* chamber. The small symbols show the mean Po values from multiple clamp protocols in ≥4 individual bilayers; for each Po value data from clamp steps to +80, +60, +40, −40, −60 and −80 mV were pooled. In the case that more than one channel was active in a bilayer we estimated the number of channels from the maximum number of simultaneous openings observed in the absence and presence of the redox compounds. The corresponding large symbols represent the mean ±SD of the independent bilayer recordings given by the small symbols. (B) Example of current fluctuations measured in the same bilayer at −80 mV in the absence and in the presence of 80 mM DTT. (C) Example of current fluctuations measured in the same bilayer at −80 mV in the absence and in the presence of 10 mM H_2_O_2_. In B and C the solid lines represent the close state of the channel. Dashed lines represent the open level of the high conductance state.

In further experiments we monitored the activity of wt and R9C mutant channels in the same bilayers before and after imposing reductive or oxidative conditions. The scatter plot in Figure 5A shows that addition of DTT or H_2_O_2_ has in the majority of experiments no consistent effect on the open probability of the wtPLN channel; the Po value is in the presence of DTT or H_2_O_2_ not significantly different from that in the control (P = 0.8 for DTT and 0.5 for H_2_O_2_ treatment). In experiments in which we measured the activity of the R9C mutant channel in individual bilayers before and after addition of DTT or H_2_O_2_ to the buffer shows that both redox agents consistently evoked an increase in channel open probability; under oxidative conditions Po quasi approaches the open probability of the wt channel (Figure 5A–C). A Kolmogorov-Smirnov test shows that the H_2_O_2_ and the DTT evoked increase in Po of the mutant channel is moderately significant (P = 0.02 for H_2_O_2_ and 0.03 for DTT). The results of these experiments imply that the mutation of an arginine to a cysteine makes the protein more sensitive to the redox environment of the buffer; any disturbance of the protein fold via reducing and oxidizing C9 in the mutant results in a modulation of the channel open probability. The data do not provide a molecular explanation for such a biphasic dependency of channel activity. But worth noting is that also the thermostability of the PLN R9C pentamer is favoured over that of the wt PLN by both oxidizing and reducing conditions[15]. In a physiological context it is interesting to note that such an optimal dependency of channel activity on the redox state was also observed for native Ca^2+^ channels in the sarcoplasmic reticulum [33].

## Conclusions

We have performed experiments which allow an estimation of the size of a channel pore from its unitary conductance [24], [26]. This analysis shows that the PLN complex, which generates channel fluctuations, has a pore size between 2.2 Å and 6.2 Å. These experimentally determined values are similar to those previously reported for the pore of the PLN pentamer on the basis of an NMR structure [9], [30] and molecular modelling [28], [29]. This good agreement between structural and functional data is a strong support for the hypothesis that the conducting channel is indeed generated by PLN in its pentameric form. The experiments further indicate that both phosphorylated and unphosphorylated PLN can generate channel fluctuations. Hence the regulatory role of PLN on SERCA activity by phosphorylation [31], [32] seems not to be related to the unitary channel conductance. The data however show that phosphorylation reduces the open probability of the PLN channel by about 50%; this small but significant effect means that over a given time less current is transported across the SR membrane. The modulation of open probability by phosphorylation is compatible with the physiological regulation of SERCA activity by pPLN. First, phosphorylation increases SERCA activity due to the known release of the interaction between monomeric PLN and SERCA. In addition the reduced open probability of the K^+^ permeable PLN channel [12] is functionally equivalent to the inhibition of K^+^ channels in the SR. The latter has been shown to increase the Ca^2+^ content in the SR probably by corrupting the charge balance system [34]. The Ca^2+^ content in the SR may also be increased because an inhibition of a K^+^ conductance in the SR can inhibit the release Ca^2+^ from the organelle [35].

This interpretation of PLN function is also in agreement with the view that the R9C mutant affects SERCA activity; the reduced open probability of this mutant mimics the PLN in its phosphorylated state. The low Po value may result in an aberrant elevated Ca^2+^ accumulation in the SR even in the absence of control by phosphorylation. Altogether it occurs that the mutation of an arginine into a cysteine is not crucial for channel formation and unitary conductance. However this mutation, which increases the stability of the pentamer [15], lowers the open probability of the PLN channel. In the context of recent computational simulations on the PLN pentamer it is reasonable to speculate that the probability of opening of the central pore is antagonized by a tight interaction between the monomers [36]. Hence the possible involvement of the R9C-PLN mutation in dilated cardiomyopathy might be explained, at least in part, by a modulation of the open probability of the PLN generated channel conductance; this would be a modulatory factor beyond the altered interaction of R9C with SERCA.

## Methods

### Wild Type PLN and mutants plasmids

The pMALc2x vector containing the wild type human PLN gene was kindly provided by Prof. J. J. Chou (Harvard Medical School, Boston, USA); it was expressed as a fusion protein with the maltose-binding protein (MBP). Wt-PLN plasmid was inserted in BL21-rosetta (Novagene, Madison, WI, USA).

Two point mutations R9C and I47A were individually introduced in the wt-PLN by the QuikChange II XL Site Directed Mutagenesis kit (Agilent Technologies, Inc., Santa Clara, CA, USA). R9C and I47A plasmids were inserted in BL21(DE3) pLysS E. coli (Promega, Madison, WI 53711 USA).

### Expression and Purification

A 10 ml starter culture containing LB growth media supplemented with 100 µg/ml ampicillin and 30 µg/ml chloramphenicol was inoculated with a single colony and agitated at 210 rpm overnight at 37°C. For expression of the MBP-PLN fusion construct, the starter culture was diluted into 1 L of LB growth media supplemented with 100 µg/ml ampicillin and 30 µg/ml chloramphenicol and grown at 37°C, 200 rpm up to an OD600∼0.6. Protein expression was induced by 0.4 mM IPTG and cells were harvested after overnight induction at 25°C. Cells were harvested by centrifugation. Cell pellets were resuspended in 30 ml lyses buffer (25 mM Tris pH 8.0, 100 mM NaCl, 1 mM EDTA, 1% Triton X-100, 10 mM dithiothreitol, 1 mM phenylmethylsulfonyl fluoride) and sonicated on ice using a Sonoplus 2070 (Bandelin electronic, Berlin, Germany). The lysate was passed through amylose resin (New England Biolabs, Ipswich, MA, USA) in order to isolate the pure MBP-PLN fusion protein. The composition of elution buffer was Tris-Base 25 mM, NaCl 100 mM, EDTA 1 mM, DTT 1 mM, Triton 0,1% and Maltose 25 mM. The fusion protein in elution buffer was cleaved overnight at room temperature with a fully active TEV protease variant with improved stability and high solubility. The vector embedding the TEV protease was kindly supplied by Prof. S. P. Bottomely (Monash University, Australia) and expressed as described in [21]. Subsequently PLN was precipitated by centrifugation in ethanol at 4°C, resolubilized in Hexafluoro-2-propanol (Sigma Aldrich, Steinheim, Germany), Formic Acid (Sigma Aldrich, Steinheim, Germany) and H_2_O at a ratio of 1∶1∶1 (2 ml per mg of fusion protein) and purified by FPLC (Akta PrimeÄKTAprime™ plus, GE Healthcare Pittsburgh, PA, USA) using a reverse-phase column C18. Proteins were lyophilized, resuspended in 50% Acetonitrile (Sigma Aldrich, Steinheim, Germany) and lyophilized again. The pure PLN fraction was identified by SDS-PAGE and MALDI-TOF and quantified by Lowry protein assay (BCAPierce, USA). K27A was obtained as described in [22]. Purity and oligomeric state of the PLN samples is shown in Figure S1.

### Planar lipid bilayer and single channel measurements

Experiments with planar lipid bilayers were carried out as described previously [23] using the folding method with a 10 mg/ml solution of diphytanoylphosphatidylcholine (DPhPC) (AvantiPolar, AL, USA) in pentane. The experimental chambers used to assemble the planar bilayer were either custom made or disposable chambers (Ionovation, Osnabrück, Germany).

The measurements were performed in a buffer containing 500 mM KCl, 10 mM Mops/Tris pH 7. The Ag/AgCl electrode in the *cis* compartment was directly connected to the head stage of a current amplifier (EPC 7, List, Darmstadt, Germany); the *trans* chamber was grounded. Currents were recorded and stored by an analogue/digital-converter (LIH 1600, HEKA electronics, Lambrecht, Germany) with a sampling rate of 3.571 kHz after low pass filtering at 1 kHz. Data were recorded by Patchmaster-Software (HEKA electronics, Lambrecht, Germany) and analyzed with the Fitmaster-Software (HEKA electronics, Lambrecht, Germany) and the KielPatch program (University of Kiel, www.zbm.uni-kiel.de/aghansen/software.html) and Origin (OriginLab. Northampton, MA, USA). The apparent single channel current amplitudes (I_app_) were determined by visual inspection of the current traces using the KielPatch software. The open probability (Po) was calculated with the KielPatch software. In the case that more than one channel was present in a bilayer we estimated the number of channels from the maximal number of concomitant open events.

The protein, which was dissolved in water, was added directly to the trans chamber at a final concentration of ca. 0.3 µM. Before addition of the protein the bilayer conductance was routinely recorded for approximately 1 hour in order to exclude artefacts from contaminations. Only bilayers without artefacts were used for reconstitution of PLN. To perform experiments of phosphorylated wt-PLN, the protein was added to the bilayer after 3 h incubation at 30°C under the following conditions: 1 mg/ml wt-PLN, 20 mM imidazole, 100 mM KCl, 1 mM DTT, 10 mM MgCl_2_, 0.5 mM EGTA, 1 mM ATP and (5 units/10 µg PLN) PKA (Sigma) diluted in storage buffer solution. Storage buffer solution consists of 20 mM imidazole/100 mM KCl with 50% glycerol. According to Glaves et al. 2011 [16] we can assume that this treatment generates a complete phosphorylation of PLN at Ser16 (see Figure S1). To examine potential artefacts from the buffer in which PLN was phosphorylated we added the complete mixture in control experiments in the absence of PLN to the bilayer. In these test experiments we did not detect any channel activity.

### Calculation of pore size

The pore size has been calculated from the electrical conductance as described by Hille [24] using equation (1):
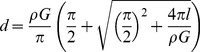
(1)where d is the diameter of the pore, G is the conductance (12 pS in 250 mM KCl symmetrical solution, Figure 1B), l is the length of the constriction zone, (assumed to be 28 Å according to the NMR structure of Oxenoid and Chou, 2005 [9]), and ρ is the resistance of the solution, 49.5 Ωcm for 250 mM KCl solution; taking into account the correction factor of Smart et al. (1997) [25] the resistance in the protein is 247.5 Ωcm [26].

### The Polyethylene glycol method

To estimate the pore size we performed experiments in the presence (20%) of non-electrolyte molecules (PEG) of different molecular weight. Non-electrolytic polymers are approximately spherical and with a defined hydrodynamic radius; the latter are reported in Krasilnikov et al. [27]. In the presence of these polymers the conductance of the bulk solution and the conductance of the channel decreases as a function of the hydrodynamic radius of the polymers. At the characteristic cut-off polymer radius the concentration of PEG in the pore is reduced to 1/e of the original concentration. The experimental data were interpreted according to [25], [26] assuming that the PLN channel has a central constriction zone, which is on both sides flanked by wide vestibules. It is assumes that the onset of exclusion of PEG from the pore is giving rise to the region where the ratio G_20_/G_0_ of conductance in the presence of polymer (G_20_) versus that in the absence (G_0_) starts to rise. The part of the curve where the ratio of G_20_/G_0_ approaches the maximal value is likely to reflect the wide end of the pore radius [25]. To obtain the respective minimal and maximal radii, the experimental data were fitted by a logistic equation (2).
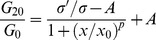
(2)were G_0_ is the conductance in 250 mM KCl solution, G_20_ is the conductance in 250 mM KCl solution plus a polymer at 20%. The ratio σ′/σ ( = 0.553) gives the relative bulk conductivities in the presence (σ′) and absence (σ) of polymer, A is the maximal value measured in polymer free solution. The value x_0_ is the point of inflection and p the power of the logistic equation. The second derivative of this curve provides an indirect measure for the radius in the narrow constriction zone and the wider pore mouth [25], [26]. Experiments with sucrose and ethylene glycol were omitted from the analysis. The former evoked spike like openings with high conductance levels and sublevels; the latter caused an anomalous increase in conductance.

## Supporting Information

Figure S1 Purity and oligomeric state of wt- and mutant PLN proteins used for reconstitution in bilayers.
(DOC)
