# Supplementary material for: Structure-Function Relation of Phospholamban: Modulation of Channel Activity as a Potential Regulator of SERCA Activity
Source: PLoS One. 2013 Jan 4;8(1):e52744. doi: 10.1371/journal.pone.0052744 (PMC3537670; doi:10.1371/journal.pone.0052744)
Supplement: Figure S1 — Purity and oligomeric state of wt- and mutant PLN proteins used for reconstitution in bilayers. (DOC) [file pone.0052744.s001.doc]

Supplemental material

**Figure S1:** Purity and oligomeric state of wt- and mutant PLN proteins used for reconstitution in bilayers.

To asses the purity of the proteins, which were used for reconstitution in bilayers, we analysed samples by SDS-PAGE at 15 or 17%. Proteins were solubilised in 1% triton.


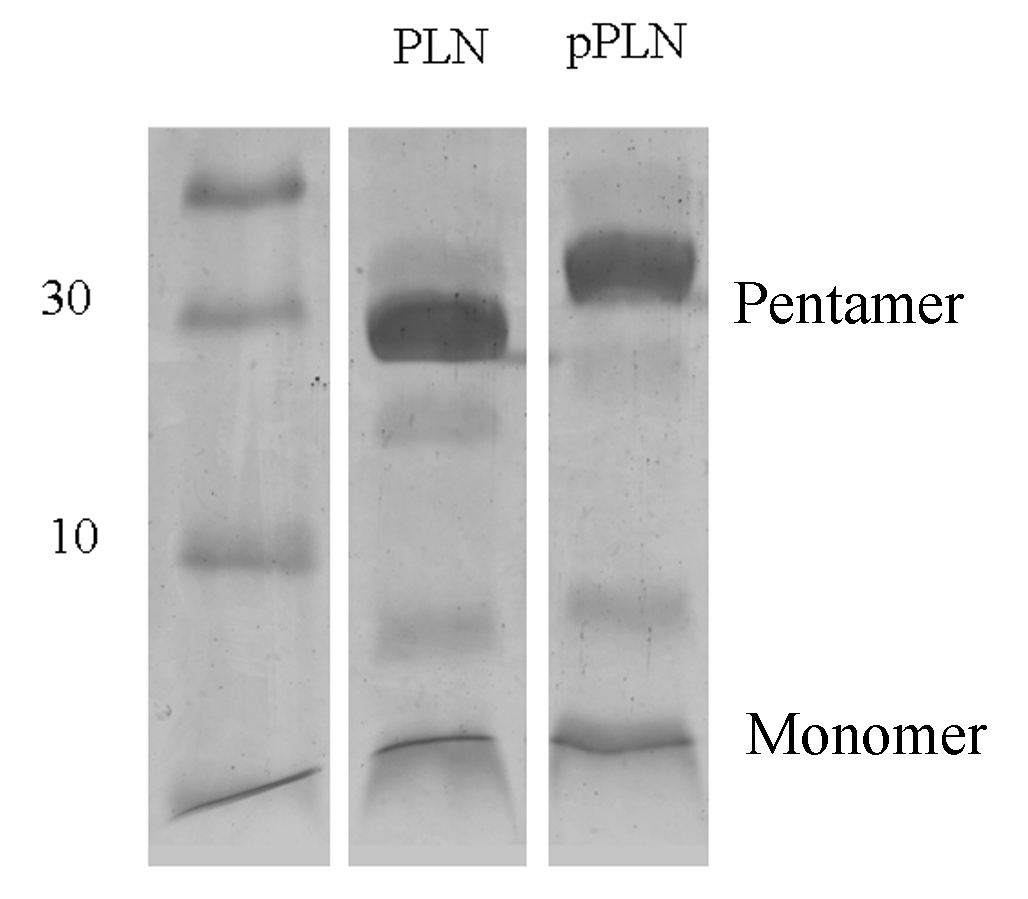


Figure S1.1: SDS-PAGE gel (15% tris-glycine) with not-phosphorylated (PLN) and phosphorylated (pPLN) wt PLN protein. The majority of the protein is in both cases pentameric. The procedure results in a proper phosphorylation of the protein.


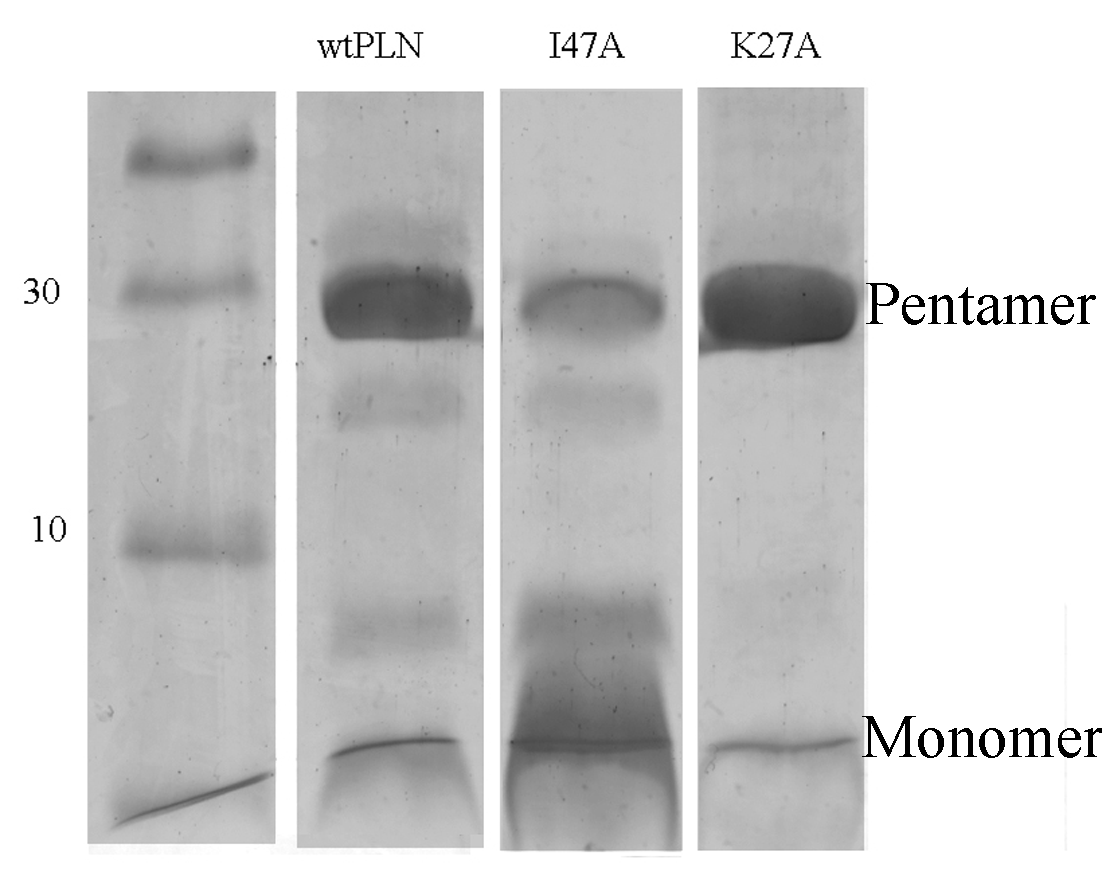


Figure S1.2: SDS-PAGE gel (15% tris-glycine ) for wt-PLN and mutants I47A and K27B. The mutation I47A decreases the pentameric and increases the monomeric form of the protein.


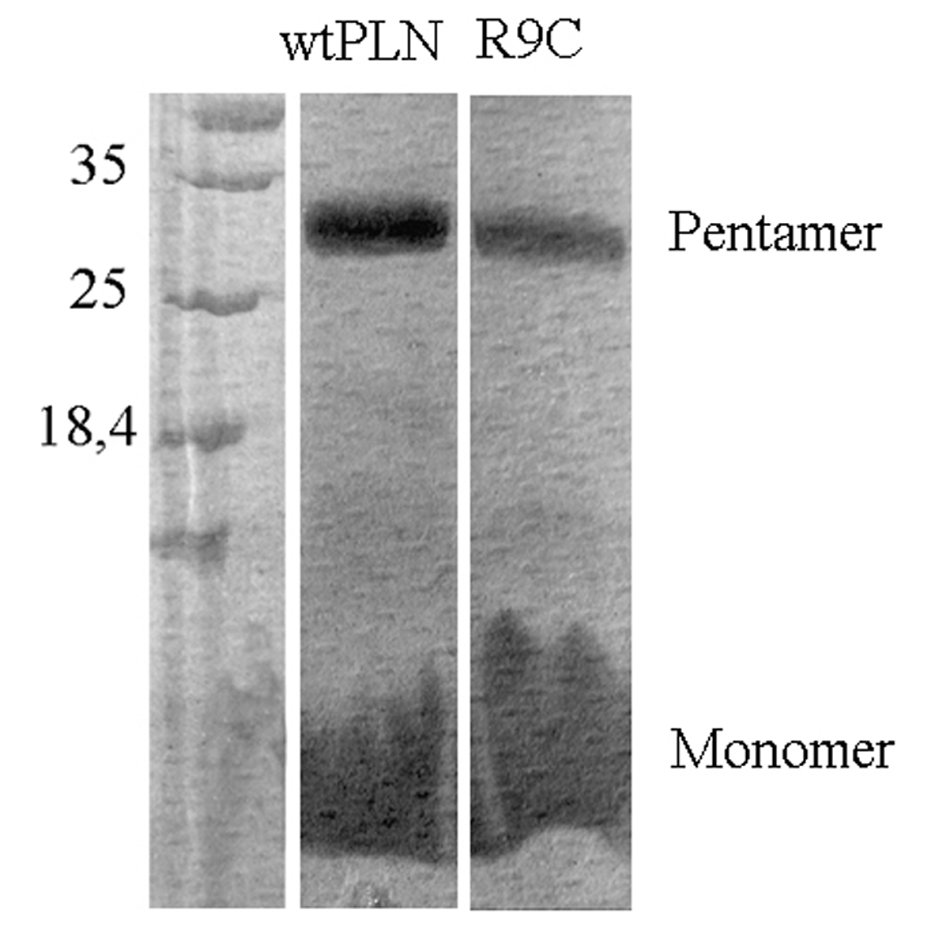


Figure S1.3: SDS-PAGE gel (17% tris-glycine) for wt-PLN and R9C mutant.
